# Defining Household Typologies Based on Cropland Use Behaviors for Rural Human-Environment Systems Simulation Research: A Case Study in Southwest China

**DOI:** 10.3390/ijerph19106284

**Published:** 2022-05-22

**Authors:** Ming Li, Yukuan Wang, Congshan Tian, Liang Emlyn Yang, Md. Sarwar Hossain

**Affiliations:** 1 Institute of Mountain Hazards and Environment, Chinese Academy of Sciences, Chengdu 610041, China; liming@imde.ac.cn; 2 College of Architecture and Urban-Rural Planning, Sichuan Agricultural University, Chengdu 611830, China; 3 Department of Geography, Ludwig-Maximilians-Universität München, 80333 Munich, Germany; emlyn.yang@lmu.de; 4 Environmental Science and Sustainability, School of Interdisciplinary Studies, University of Glasgow, Dumfries DG1 4ZL, UK; mdsarwarhossain.sohel@glasgow.ac.uk

**Keywords:** household typology, multivariate analysis techniques, household decision-making, agent-based model, rural environment protection

## Abstract

The dynamics of rural human-environment systems in developing countries have increasingly been attracting attention. Agent-based modeling (ABM) is a valuable simulation tool for detecting complex feedback loops in rural human-environment systems with a ‘bottom-up’ approach. However, such models require the prerequisite analysis of household typology to simulate households’ decision-making process, where a gap exists between having accurate classification criteria and a simplified modeling framework. This study aimed to develop a household typology for two selected counties in southwest China based on multivariate analysis techniques and the classification tree method. Four categories of socioeconomic variables, including labor conditions, resource endowments, economic status, and social connections, were screened as possible factors impacting agriculture practice decisions. The results showed that household diversification in the study area was mainly determined by diversified livelihood strategies of off-farm work, livestock breeding, subsidy dependence, and traditional planting. Five distinct household types were identified: non-farm households, part-time households, livestock breed households, subsidized households, and traditional planting households. The household types were associated with specific cropland use behaviors, and their decision-making behaviors were verified with bounded rationality theory (where the maximization of profits is the primary goal). The quantitative classification criteria obtained in this study were clear and could be easily identified and used by ABMs. Our study provides a basis for further simulation of the complicated rural human-environment systems in southwest China.

## 1. Introduction

Currently, China is putting significant efforts into solving its environmental problems while reducing poverty in especially rural areas [1,2]. It is well known that current environmental problems in rural China mainly come from cropland, because forests and grasslands have been effectively protected under the Grain for Green Project and the Natural Forest Protection Project [3,4]. Some environmental problems, including soil erosion and non-point source pollution, are generated during agricultural production [5,6], and differentiate greatly across cropland use intensities [6,7] and households living styles [8]. Household decision-making behavior plays a decisive role in this process; thus, it is essential to explore the impacts of household decision-making behavior on cropland uses in rural environment studies [6].

Agent-based modeling (ABM) with its ‘bottom-up’ approach is increasingly used as a powerful tool for modeling complex changes in rural human-environment systems [9,10]. The core of ABMs is to simulate the decision-making process of individual agents and their interactions with each other and with the environment [11,12,13]. Households are recognized as the most important agent types in rural human-environment systems simulation research based on ABMs, and the accuracy of model simulation is often affected by the complexity of households’ decision-making behaviors [13]. Linear programming models are effective ways to simulate the complex processes of households’ decision-making with limited survey samples [14]. However, aggregation bias is a common problem in these household level modeling methods [15], which require appropriate classification of household types [16]. In addition, the simulation of households’ decision-making in ABMs is based on the conversion of agent types and the assumption of path dependence [13,17]. Thus, agent typology analysis is a prerequisite for ABM studies [12,16]. 

Several studies have used various criteria and methods to describe diversity in rural households or farmers, which varies greatly depending on the purpose of the classification. Some authors qualitatively defined household types based on a single criterion or several criteria. For example, the proportion of non-farm income was used in China to study household livelihoods status and its influence on regional land use change [18]. Baccar et al. (2017) classified family farms in Morocco into four types based on combinations of three criteria, including access to land, production choices, and access to water, and identified their development pathways from an initial similar situation to a contemporary diverse situation [19]. In other studies, multiple criteria combined with the multivariate analysis techniques were used to differentiate rural households or farmers quantitatively. For example, Tittonell et al. (2010) tried to describe rural livelihood diversity and its influence on soil fertility in East Africa using resource endowment and income strategies’ indicators in a principal component analysis (PCA) [20]. Dossa et al. (2011) screened variables for farm activities, farm resource endowment, and production orientation to explore household diversity in Sudano-Sahelian West Africa, applying PCA and cluster analysis (CA) [21]. Some current studies recommend combining qualitative and quantitative approaches in a complementary manner to take advantage of their meticulousness and objectivity [22,23]. However, there is still a gap between having quantitative classification criteria and a simplified classification framework.

Most ABM studies classified household types using empirical methods for the purpose of model simplification, but few of them tried to differentiate households quantitatively. For example, farmers in the American Corn Belt were classified into four types based on factors influencing their conservation practice decision-making using a simple mutually exclusive method [12]. Household livelihoods in the semi-arid region of Northeast China were classified into three types according to different strategies for simulating local economic structural change [24]. Valbuena et al. (2008) applied a ‘classification tree’ to simulate land use/cover change (LUCC) driven by local decisions, using farmers’ views, farm characteristics, and location [16]. Farmers in East Lesvos, Greece were classified into three types based on individual ability and willingness to farm using cluster analysis to describe the influence of cultural and behavioral transformations on landscape futures [13]. 

Our literature review found that for the purpose of ensuring that each type of household agent has significant differences in their decision-making, and obtain relatively simple classification criteria as required by the ABM simulation, combined qualitative and quantitative typology analysis is needed based on the characteristics of or the factors impacting household agricultural production decisions. Thus, the objectives of this study were (a) to present an application of multivariate analysis techniques and the classification tree method for household typology identification, (b) to evaluate the reliability of the method by conducting statistical tests on their actual cropland management behaviors within-group variance, and (c) to discuss strengths and weaknesses of the method used for rural studies, especially ABM studies. 

## 2. Material and Methods

The dataset used in this study was derived from a baseline survey in two Southwestern China counties of Baoxing and Puge, designed to characterize the plot-scale differences of cropland disturbance intensity and its environmental consequences [6,7].

**Figure 1:**
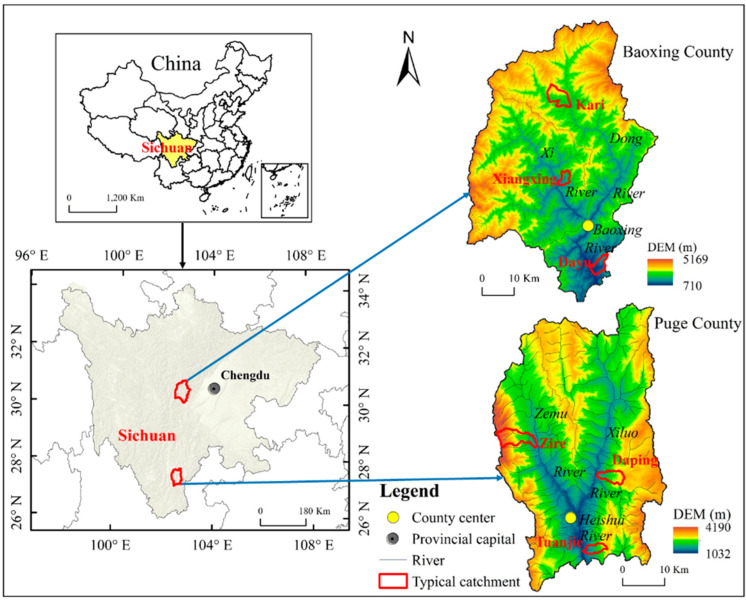
Location of the study areas.

Baoxing county is located at 102°28′–103°02′ E and 30°09′–30°56′ N, has an area of 3144 km^2^, had an estimated population of 59,000 inhabitants and 21,500 households at the end of 2014, and the population density was approximately 19 per square kilometer [6]. Puge county is located at 102°26′–102°46′ E and 27°13′–27°30′ N, has an area of 1905 km^2^, had an estimated population of 199,000 inhabitants and 56,000 households in 2014, and the population density was approximately 104 per square kilometer [7]. Baoxing is one of the most important panda habitats in China, and is one of the world’s 25 ‘biodiversity hotspots’ [25]. Puge is located in the upper reaches of the Jinsha River Basin, and faces extreme soil water erosion and multiple natural hazards [26] (Figure 1). There are challenges to effective ecological and environmental restoration in both counties.

### 2.1. Data Collection

About 10% households of the six sample villages were randomly selected and surveyed based on the stratified random sampling method. Finally, valid questionnaires were 78 in Baoxing and 249 in Puge, with an effective rate of 95% and 97%, respectively. Household surveys were performed from July to September 2015 using a structured questionnaire. The questionnaire was designed with reference to the Sustainable Livelihoods Framework to understand how household-level socioeconomic factors affect households’ cropland use decisions. The following information was included in the questionnaire: possession of different kinds of livelihood assets, off-farm work, consumption, cash income, animal production, and detailed agricultural production processes. Data were collected via face-to-face interviews with householders, and this process took about 2–3 h.

### 2.2. Development of Household Types

#### 2.2.1. Variables Selection

When defining typologies, the variables selected for their construction are more important than the classification technique applied [12]. Variables should be selected based on the specific objectives of the classification, and only indicators that have significant impacts on household differentiation should be included. 

Many studies show that cropland use is significantly affected by a household’s labor conditions, resource endowments, economic conditions, and social connections. For example, Burton (2014) reviewed the complex causality between household’s demographic characteristics and their environmental behaviors [27]. Zhang et al. (2014) revealed that family labor force characteristics are important factors influencing cropland abandonment, and thus directly affects cropland use intensity [28]. In this study, we screened six household labor force indicators including number, age, and education to define household typologies, namely agricultural labor scale, rate of female labor, rate of off-farm labor, agricultural labor age, household head education, and household labor education.

Agricultural system diversity is also deeply affected by household resources endowments [21], which in turn affect agricultural production intensity [29]. In this study, five indicators including household’s farmland area, cultivated land area per agricultural labor, forest area, SLCP land ratio, and abandoned farmland area ratio were screened to reflect the influence of resource endowments on a household’s cropland use behaviors. 

Economic conditions and income strategies reflect differences in farmers’ purchasing power for agricultural materials, which directly affect agricultural production decisions [30,31]. Indicators of cash income, household deposits, access to cash credit, and the composition of the family farm’s income were considered in this study. Besides, a household’s social connections to the outside world may also have a decisive influence on their agricultural production decision-making [6]. Thus, two indicators, including if they participated in professional agricultural cooperative organizations and the number of relatives in the village, were considered in this study. Based on the above analysis, household typology delineating variables screened in this study are shown in Table 1.

#### 2.2.2. Methods for Typology Classification

Several multivariate techniques are frequently applied for classification, including Factor Analysis (FA), Multidimensional Scaling (MDS), Principal Component Analysis (PCA), and Cluster Analysis (CA). PCA and CA methods are commonly combined to carry out quantitative classification research in rural areas [21,32,33]. However, to use the standard PCA appropriately, it needs to satisfy the basic assumptions that all variables are continuous and that there is a linear correlation between them [34]. Hierarchical and K-means clustering methods are the two most widely used clustering methods [32,33,35]; however, they are not suitable for use with variables of mixed measurement levels [36]. To overcome these limitations, Categorical Principal Component Analysis (CATPCA) and two-step clustering were used in this study. The classification tree method was further used as a qualitative tool for the final household agent definition.

1. CATPCA for dimensionality reduction
  Because the CATPCA method can handle nominal, ordinal, or numerical variables simultaneously, and can also apply to non-continuous variables [37], it was used in this study to reduce the original set of 21 indicators into a smaller number of components. To obtain the reliable number of principal components, components that contain at least four variables having a loading score > 0.50 should be retained [21,38]. CATPCA component loading maps were used to predict the number of homogeneous household groups. The selected variables with high loading scores were subsequently used as inputs for the cluster analysis.
2. Two-Step clustering method for household classification
  The basic assumptions for the two-step clustering method are that continuous variables must be normally distributed and categorical must be multi-nominally distributed; and variables must be independent [39]. Fortunately, the two-step cluster procedure of SPSS is fairly robust to violations of the two assumptions [40], thus making it a suitable clustering method for multi-dimensional data sets [21,37]. The selected continuous variables were converted into nominal ones before using this procedure for overcoming the categorical and continuous variable weight misallocation [21]. Cluster analysis validation results were graphically shown using SPSS, where a cluster cohesion and separation value > 0.7 indicated excellent separation; 0.5~0.7 indicated a clear assignment of data points to cluster centers; 0.25~0.5 indicated the unclear assignment of many data points; and values < 0.25 indicated that it was practically impossible to find a significant cluster.
3. Classification tree for obtaining classification criteria
  A ‘classification tree’ was chosen as the most appropriate method to construct the typology in this study. Based on Boolean statements, indicators with the most obvious differences between clusters were used as classification criteria in the classification tree method to construct typologies. The quantitative classification criteria were based on the results of CATPCA, two-step cluster analysis, and existing qualitative classification criteria [18,41]. Names were assigned to the final household types based on their specific profiles.

### 2.3. Analysis of Cropland Use Behaviors between Household Types

In order to verify the effectiveness of our household typology, differences in households’ actual cropland management behaviors were analyzed. Cropland use behaviors were separated into physical and chemical disturbance behaviors [6] (Table 2), which reflected the potential risks of rural soil erosion and non-point source pollution, respectively. We used the Kruskal–Wallis test to explore differences within the identified groups on their cropland use behaviors. Besides, crops were separated into food crops and cash crops to reflect the possible differences on decision making goals regarding different household types.

## 3. Results

### 3.1. Household Types Classification

#### 3.1.1. Interpretation of the CATPCA

**Figure 2:**
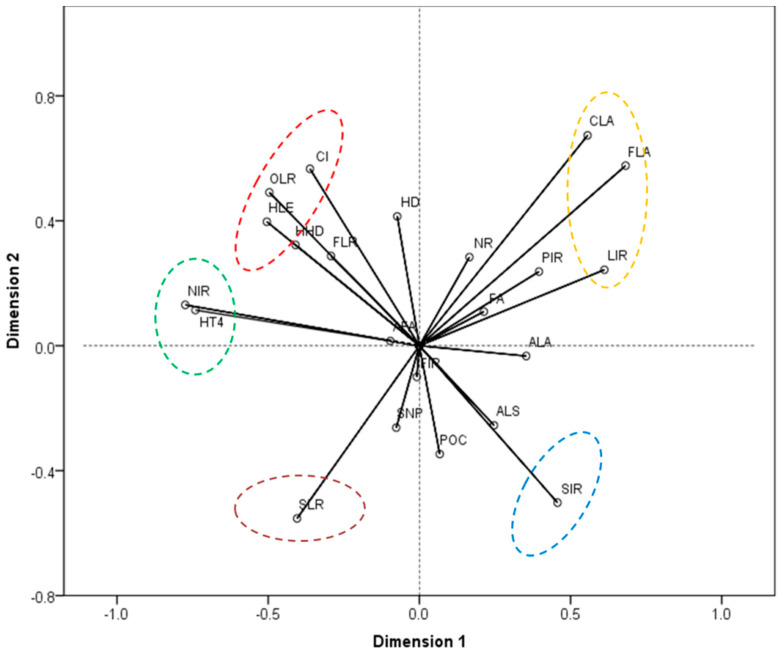
Component loading plot obtained from CATPCA describing the relationships between household characteristics.

The variables related to factors influencing household decision-making were distributed into two components by CATPCA (Table 3). The Total Cronbach’s Alpha was 0.885; the Total Eigenvalue was 6.358; and the Total variance was 30.277%. All parameters indicated a good model fit. Two-dimensional component loading plots obtained using CATPCA are shown in Figure 2, which summarizes the relationships between the different variables. The most influential variables were identified based on the length of the vectors, and were related to household labor conditions (HLE), resource endowments (FLA, CLA, and SLR), and economic conditions (CI, NIR, LIR, and SIR). In addition, Figure 2 suggests that five a priori household groups could be identified in the study area, according to the relationships among the most influential variables.

#### 3.1.2. Two-Step Clustering Analysis

The Two-Step cluster algorithm also suggested a five-cluster solution (Table 4). The quality of the clustering solution was good with cluster cohesion and a separation value of 0.55. The top three variables with the largest predictor importance values were SIR, LIR, and NIR (Table 4). The distribution of the identified household clusters is shown in Table 4.

#### 3.1.3. Classification Criteria by Classification Tree

**Figure 3:**
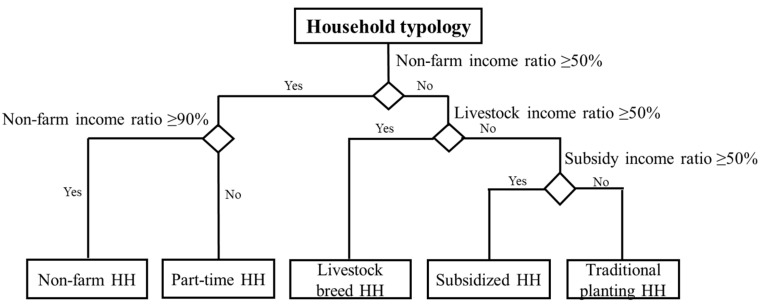
Household type definition. Note: ‘HH’ is the abbreviation of ‘household’.

The classification tree method was used to obtain quantitative classification criteria based on Boolean statements defined by the top three variables: NIR greater than 50% and 90%, LIR greater than 50 %, and SIR greater than 50%. The quantitative classification criteria were supported by the results from the CATPCA, two-step cluster analysis (Table 4), and existing qualitative classification criteria [18,41], in which the indicator of non-farm income ratio > 60% and non-farm income ratio > 90% were used to identify non-farm household and non-farm household types. Finally, five household types were obtained and were assigned the following names: non-farm households, part-time households, livestock breeding households, subsidized households, and traditional planting households (Figure 3).

Our results showed that household types in the Baoxing County were mainly part-time and non-farm households, accounting for 32.3% and 25.7%, respectively. In Puge County, on the other hand, traditional planting household dominated, accounting for 53.8% (Table 5).

### 3.2. Description of Household Types

**Figure 4:**
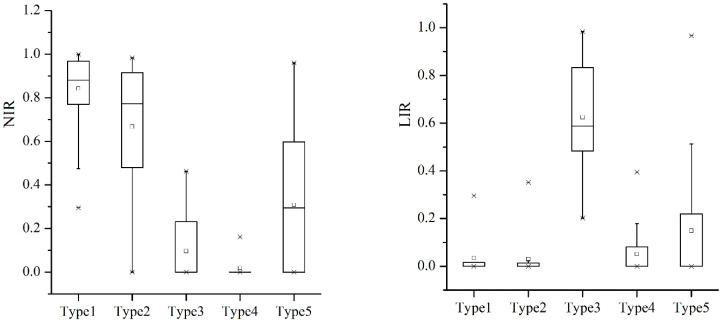

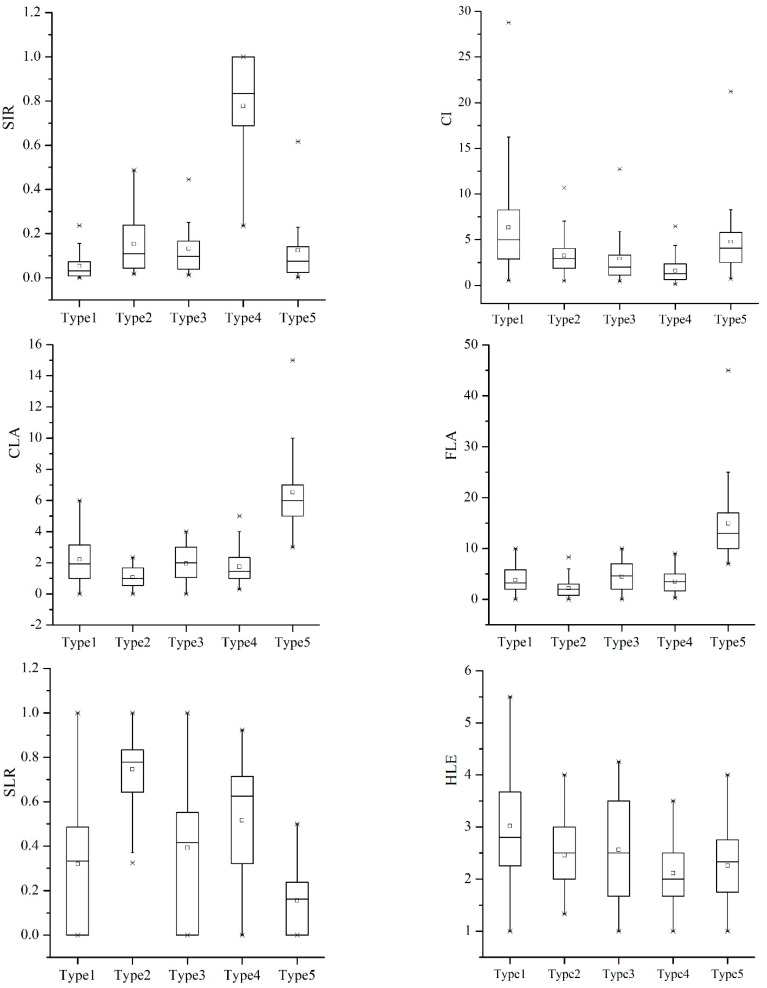
Description of household profiles. Boxplots represent the mean (black point), median (solid lines), first and third quartiles (contained in the boxes), dispersion (dashed line), and outliers (asterisk) of the distribution of the ranks of each trait. Notes: Type 1, non-farm households; Type 2, part-time households; Type 3, livestock breed households; Type 4, subsidized households; Type 5, traditional planting households.

Specific profiles of each household type were described with the eight most influential variables and are presented in Figure 4.

**Non-farm households (type 1).** These households had the highest level of cash income, with the highest NIR and the lowest LIR and SIR. The labor force in these households had much higher education than in other households and were usually more inclined to take part in the Grain for Green Project, as confirmed by their high level of SLR.

**Part-time households (type 2).** These households had the second highest CI levels in the study area, and NIR was second only to the non-farm households. In most cases, the young and middle-aged labor force engaged in non-farm work, whereas the old labor force engaged in planting at home. Resource endowments in these households were the lowest, with the lowest levels of CLA and FLA, and they were more inclined to take part in the Grain for Green Project, with the highest level of SLR.

**Livestock breed households (type 3).** These households had the second lowest CI levels in the study area. Livestock breeding was the basic livelihood strategy, and they generally did not consider non-farm work, as verified by their low level of NIR.

**Subsidized households (type 4).** These households had the lowest CI levels in the study area, with the highest proportion of subsidy income. Labor conditions in these households were the poorest, with the smallest labor force and the lowest education level. Traditional planting and breeding were also their important sources of livelihoods beside subsidies. Due to the limited labor force, their willingness to participate in the Grain for Green Project was also very strong, as indicated by the second highest level of SLR.

**Traditional planting households (type 5).** These households had the highest resource endowments in the study area, as reflected by the highest levels of FLA and CLA. Coupled with their low level of SLR, this reflected that their livelihoods were more dependent on traditional planting. Because cash crop planting was their main livelihood strategy, their cash income levels were not as low as expected.

### 3.3. Cropland Management Behaviors of Each Household Type

#### 3.3.1. Cropland Physical Disturbance Behaviors

The identified household types were associated with specific cropland physical disturbance behaviors, and generally showed the same trends between planting food crops (corn) and cash crops (tobacco). The statistically significant descriptors are shown in Table 6. The livestock breed and traditional planting households had higher tillage method (TM) scores, reflecting their broad usage of conventional tillage, whereas subsidized households were more likely to engage in reduced or no tillage. The differences in manure conservation (MC) and fertilization method (FM) between household types were interesting. Non-farm households were related to lower MC and higher FM, whereas the livestock breed households were related to higher MC and lower FM. This pattern indicated that non-farm households were more inclined to use inorganic fertilizers with deep fertilization, whereas the livestock breed households were much more in favor of using farmyard manure with broadcast fertilization. Finally, manual weeding practices were mainly adopted by the subsidized households, whereas few other types (especially the non-farm, part-time, and livestock breed households) followed such a practice.

#### 3.3.2. Cropland Chemical Disturbance Behaviors

The identified household types identified were associated with specific cropland chemical disturbance behaviors and showed different trends between food and cash crops. The statistically significant descriptors are shown in Table 7. The indicator nitrogen input from fertilizers (NIF) reflected the actual inorganic fertilizers’ usage of each household type. Non-farm and part-time households had fairly high NIF levels for food crops planting, whereas traditional planting households had the highest value for cash crops planting. The same patterns were observed for the indicator of total nitrogen input (NI). Pesticides’ costs in both cases also showed different trends. For food crops planting, non-farm and part-time households had much higher pesticide usage (PU) values, whereas livestock breed households had the lowest values. With respect to cash crops planting, part-time, livestock breed, and traditional planting households had much higher levels, whereas non-farm and subsidized households had lower PU values. Finally, in both cases, herbicides were used the most by part-time households and least used by subsidized households.

## 4. Discussion

### 4.1. Household Types

Many household-level socioeconomic factors have been found to be related to household decision-making behaviors. However, for simulating the complex changes to rural human-environment systems using ABM, indicators used for the classification of households should be as simple as possible [16]. In this study, we generated household typologies using the top three of 21 screened indicators: non-farm income ratio (NIR), livestock income ratio (LIR), and subsidy income ratio (SIR) (Figure 3). The CATPCA data reduction technique was combined with two-step clustering analysis and the classification tree method.

Cluster analysis is an effective method for typology studies [15,21,22,23,33,42]. The identification of key variables and the determination of the most suitable number of clusters are the two core issues when using cluster analysis methods [21]. In this study, CATPCA was used as the data reduction technique for dealing with variables of mixed measurement levels and predicting the number of homogeneous groups. The five household groups determined by CATPCA were then confirmed using the two-step cluster analysis method. Dossa et al. (2011) reported six different urban and peri-urban agriculture systems in three West African cities when applying similar classification methods to those that we used in our study [21]. Different cluster solutions were also reported by Li et al. (2012) when using a non-hierarchical clustering approach to reflect household livelihood diversification in China [43].

As opposed to the current methods, however, we obtained classification criteria based on the classification tree method with reference to the top three indicators identified by two-step clustering analysis (Figure 3). The application of joint objective methods and subjective judgment made the typology analysis more reliable and applicable, which has also been confirmed by many scholars [15,21,23,42]. The classification criteria for household types obtained in this study were clear and easy to be identified by the ABMs.

Based on the current knowledge, we screened 21 social-economic indicators that potentially influenced households’ cropland use behaviors (Table 1). However, only eight indicators were identified as the most influential variables by the CATPCA method, and half of them belonged to the economic conditions category (Table 3, Figure 2). These results were consistent with previous findings showing that household livelihood strategies, especially for non-farm work, had a direct influence on farming behavior [6,18,44]. Interestingly, the top three variables identified by the two-step cluster analysis method and used for the final typology classification (Figure 3) belonged to the economic conditions category. To some extent, this finding also supports the traditional classification of households in China by using the single criterion of non-farm income [18,41] and the multi-criteria of livelihood assets [45]. However, our classification methods can successfully differentiate the full-farm household type in previous study [18,41] into the livestock breed households and traditional planting households, and identify a fully new household type of subsidized households. The significant differences among their cropland use behaviors (Table 6 and Table 7) verified the necessity and effectiveness of our household typology.

### 4.2. Household Types and Cropland Use Behaviors

Other research shows that cropland use behaviors in Table 2 can be differentiated as labor-saving (PU, HU, and TM), yield-increasing (NIF, NIM, and NI), and environment-protecting (FM, MC, and MW) inputs [46]. Our results showed that for food crops planting, non-farm and part-time households had much higher labor-saving and yield-increasing input levels, with lower levels of an environment-protecting input, as verified by their higher score of NIF, NI, PU, HU, and FM (Table 6 and Table 7). This was likely because non-farm and part-time households were more likely to engage in non-farm employment with labor recovered by their much higher purchasing power of agrochemicals (including NIF, HU, and PU). However, since they remained dependent on agricultural production [6], it was also necessary for them to enhance yield-increasing inputs to ensure food crop yields. These findings supported the bounded rationality theory of household decision-making, which states that households’ behaviors are based on the weighing of multiple goals of profit maximization, risk aversion, and leisure [47,48]. The other three types of households were more likely to apply conventional tillage, farmyard manure, and manual weeding to reduce costs while ensuring crop yields [7], which explained why they had higher levels of environment-protecting inputs.

For cash crops planting, the opposite trend was observed within the identified five household types. Traditional planting households had higher yield-increasing inputs; part-time households had higher labor-saving inputs; and subsidized households had the lowest labor-saving and yield-increasing inputs (Table 6 and Table 7). Because cash crops planting is the main livelihood strategy of traditional planting households, they were much more willing to increase yield-increasing inputs of cash crops. However, non-farm and part-time households were more inclined to reduce the planting and investments in cash crops when they realized that the benefits of planting cash crops were not as good as benefits from engaging in non-farm activities [32]. They chose to focus on non-farm activities on the precondition of planting some food crops as basic rations. These results also reflected the bounded rationality of household decision-making, with the foremost goal of maximum profit [48].

### 4.3. Implications for Further Research

For further ABMs studies, the household types obtained here can be used as a state variable of household agents, which on the one hand determines how household agents differentially allocate their labor force to each livelihood strategy, on the other hand reflects their disparate willingness to abandon or use cropland. Because labor force allocation may be different each year due to market fluctuations and policy changes [13], regional household types will be also changed dynamically. That means the state variable of household agents in the ABM model varies with model simulation time based on our quantitative classification criteria, and according to changes in each household’s income structure. The above processes make up the core mechanisms of simulating rural environmental changes based on the ABM method.

The typology methods used in this study enabled the identification of household groups, and the Kruskal–Wallis test showed that most variables representing household cropland use behaviors differed significantly among the identified groups. These indicated that the identified household types were distinct and had different agricultural production decisions. Because it was based on a reduced set of variables and a concise classification criterion, it was also easily used through the rapid household questionnaire survey with a limited set of questions. Thus, our typology can be used not only in future ABM studies, but in further studies exploring the diversity of rural household livelihoods and the dynamic changes in households’ environmental behaviors. It should be noted that the identified household types may differ greatly because of the varied purpose of classification. In addition, the actual household types in different regions may vary greatly due to different rural development backgrounds. In this regard, this study can also be viewed as an exploratory methodology that could be improved upon in additional studies.

## 5. Conclusions

This study generated a household typology in typical counties of Southwest China to simulate the complex processes of agent decisions in ABM, using the combined methods of multivariate analysis and classification trees. Household agents in the study area were classified into five different groups based on the top three household socioeconomic variable indicators. The classification criteria were clear and could be easily identified and used by the ABM. The identified household types were associated with specific cropland use behaviors, and their decision-making behaviors were verified following the bounded rationality theory with profit maximization as the primary goal. Our study thus provides a basis for the further simulation of the complicated rural human-environment systems using ABM methods and offers a framework for the further study of rural livelihoods and environmental protection. However, the actual household types in different regions may vary greatly due to different rural development backgrounds; thus, this study should be regarded as a methodology analysis that could be used in other regions rather than using the quantitative classification criteria directly.

## Figures and Tables

**Table 1:** Variables delineating household typology.

Category	Variables	Description	Mean ± SD
Labor conditions	ALS	Agricultural labor scale (in number, calculated by applying conversion factors to male and female household members in different age groups).	2.16 ± 0.88
	RFL	Rate of female labor (in %)	0.60 ± 0.23
	ROF	Rate of off-farm labor (in %)	0.30 ± 0.26
	ALA	Agricultural labor age (in years)	50.06 ± 10.27
	HHE	Household head education (1 = primary school and below; 2 = junior high school; 3 = senior high school; 4 = college and above.)	2.41 ± 1.02
	HLE	Household labor education (1 = primary school and below; 2 = junior high school; 3 = senior high school; 4 = college and above.)	2.58 ± 0.87
Resource endowments	FLA	Farmland area (in ha)	4.50 ± 4.93
	CLA	Cultivated land area per agricultural labor (in ha/person)	2.18 ± 2.15
	FA	Forest area (in ha)	36.64 ± 54.32
	SLR	SLCP Land ratio (in %)	0.48 ± 0.33
	AFA	Abandoned farmland area ratio (in %)	0.02 ± 0.08
Economic conditions	CI	Cash income (in 10,000 Yuan ^a^)	41.42 ± 39.25
	HD	Household deposits (1 = 5000 Yuan and below; 2 = 5000–10,000 Yuan; 3 = 10,000–20,000 Yuan; 4 = 20,000 Yuan and above.)	1.63 ± 1.11
	POC	Can the HH get access to cash credit? (yes, no)	1.19 ± 0.39
	NIR	Non-farm income ratio (in %)	0.54 ± 0.39
	PIR	Planting income ratio (in %)	0.09 ± 0.19
	LIR	Livestock income ratio (in %)	0.14 ± 0.24
	FIR	Forestry income ratio (in %)	0.03 ± 0.12
	SIR	Subsidy income ratio (in %)	0.20 ± 0.26
Social connections	SNP	Is the HH involved in professional agricultural cooperative organizations? (yes, no)	1.90 ± 0.30
	NR	Number of relatives in the same village.	6.65 ± 7.02

Note: ^a^ 1 USD = 6.16 yuan (during the study period).

**Table 2:** Indicators of cropland management behaviors used for exploring differences within the identified household groups.

Indicator	Definition
Cropland physical disturbance behaviors	
Tillage method (TM)	Total score of conventional tillage (CT), reduced tillage (RT), and no tillage (NT) during one crop rotation. (CT = 0.65 *, RT = 0.35 *, NT = 0 *)
Manure conservation (MC, (t ha^−1^))	Farmyard manure usage per hectare for each plot during one crop rotation.
Fertilization method (FM)	Total score of broadcast fertilization (BF) and deep fertilization (DF) applied to each plot during one rotation. (BF = 0.35 *, DF = 0.65 *)
Manual weeding (MW, day ha^−1^)	Number of manual weeding days per plot during one crop rotation.
Cropland chemical disturbance behaviors	
Nitrogen input from fertilizers (NIF, kg ha^−1^)	Total input of exogenous N from inorganic fertilizers per hectare during one crop rotation.
Nitrogen input from farm manure (NIM, kg ha^−1^)	Total input of exogenous N from farm manure per hectare during one crop rotation.
Total nitrogen input (NI, kg ha^−1^)	Total input of exogenous N from inorganic fertilizers and farm manure per hectare during one crop rotation.
Pesticide usage (PU, yuan ha^−1^)	Pesticides costs per hectare and per plot during one crop rotation.
Herbicide usage (HU, yuan ha^−1^)	Herbicides costs per hectare and per plot during one crop rotation.

Notes: * Derived from experts’ judgment.

**Table 3:** CATPCA component loading results for the study area.

Dimension	ALS	FLR	OLR	ALA	HHE	HLE	FLA	CLA	FA	SLR	AFA
1	0.247	−0.293	−0.496	0.353	−0.409	−0.505	0.682	0.556	0.213	−0.405	−0.096
2	−0.255	0.287	0.491	−0.033	0.323	0.397	0.577	0.673	0.109	−0.553	0.015
**Dimension**	**CI**	**HD**	**POC**	**NIR**	**PIR**	**LIR**	**FIR**	**SIR**	**SNP**	**NR**	**-**
1	−0.362	−0.073	0.067	−0.774	0.395	0.612	−0.009	0.457	−0.077	0.166	-
2	0.566	0.414	−0.347	0.131	0.237	0.243	−0.1	−0.502	−0.262	0.284	-

Notes: Abbreviations are: ALS—Agricultural labor scale; RFL-Rate of female labor; ROF-Rate of off-farm labor; ALA—Agricultural labor age; HHE- Household head education; HLE—Household labor education; FLA—Farmland area; CLA—Cultivated land area per agricultural labor; FA—Forest area; SLR—SLCP Land ratio; AFA—Abandoned farmland area ratio; CI—Cash income; HD—Household deposits; POC—Access to cash credit; NIR—Non-farm income ratio; PIR—Planting income ratio; LIR—Livestock income ratio; FIR—Forestry income ratio; SIR—Subsidy income ratio; SNP—Participation in agricultural cooperative organizations; NR—Number of relatives in the same village.

**Table 4:** Distribution of household clusters derived from the Two-Step cluster analysis.

	Percentage of Households		Mean ± SE		
	Baoxing (*n* = 78)	Puge (*n* = 249)	NIR	LIR	SIR
Cluster 1	21.29	8.12	0.89 ± 0.01	0.01 ± 0.00	0.08 ± 0.01
Cluster 2	34.94	17.24	0.75 ± 0.02	0.07 ± 0.01	0.06 ± 0.01
Cluster 3	18.47	7.35	0.13 ± 0.03	0.57 ± 0.04	0.13 ± 0.02
Cluster 4	8.84	11.58	0.13 ± 0.04	0.11 ± 0.03	0.53 ± 0.05
Cluster 5	16.47	55.71	0.17 ± 0.03	0.24 ± 0.01	0.33 ± 0.06
Total	100.00	100.00	0.54 ± 0.02	0.14 ± 0.02	0.20 ± 0.02

**Table 5:** Distribution of households by classification criteria from the classification tree method.

Household Typology	Percentage of Households	
	Baoxing (*n* = 78)	Puge (*n* = 249)
Non-farm HH	25.7	9.0
Part-time HH	32.3	17.9
Livestock breed HH	12.8	9.4
Subsidized HH	11.5	9.8
Traditional planting HH	17.7	53.8
Total	100.0	100.0

**Table 6:** Descriptive statistics (mean ± sd) of cropland physical disturbance behaviors in each of the five household types.

	Household Type				
	Type 1	Type 2	Type 3	Type 4	Type 5
Food crops (corn) planting					
TM	0.53 ^ae^ ± 0.13	0.53 ^a^ ± 0.15	0.62 ^ac^ ± 0.09	0.50 ^a^ ± 0.15	0.61 ^e^ ± 0.10
MC	5.21 ^ac^ ± 4.85	7.81 ^b^ ± 5.29	17.19 ^bc^ ± 6.73	14.57 ^a^ ± 7.34	11.99 ^a^ ± 7.08
FM	1.39 ^a^ ± 0.45	1.27 ^a^ ± 0.46	1.04 ^a^ ± 0.33	1.18 ^a^ ± 0.38	1.12 ^a^ ± 0.33
MW	3.93 ^ad^ ± 1.92	4.34 ^d^ ± 4.40	5.79 ^cd^ ± 4.56	29.74 ^bd^ ± 11.34	10.71 ^ae^ ± 1.85
Cash crops (tobacco) planting					
TM	0.65 ^ad^ ± 0.00	0.65 ^a^ ± 0.00	0.65 ^c^ ± 0.00	0.53 ^d^ ± 0.10	0.54 ^a^ ± 0.15
MC	2.50 ^ac^ ± 1.50	3.85 ^a^ ± 1.23	6.28 ^c^ ± 1.53	4.22 ^a^ ± 1.36	5.02 ^a^ ± 3.56
FM	0.65 ^a^ ± 0.00	0.65 ^a^ ± 0.00	0.65 ^a^ ± 0.00	0.63 ^a^ ± 0.12	0.68 ^a^ ± 0.13
MW	21.19 ^ae^ ± 8.75	30.77 ^a^ ± 6.42	23.99 ^c^ ± 9.33	36.27 ^cd^ ± 6.54	47.52 ^e^ ± 12.48

Notes: The superscripts indicate significant between-group differences (Kruskal-Wallis test, *p* ≤ 0.05). Abbreviations are: TM, tillage method (total score of conventional tillage, reduced tillage, and no tillage during one crop rotation); MC, manure conservation (farmyard manure usage per hectare); FM, fertilization method (total score of broadcast fertilization and deep fertilization); MW, manual weeding (number of manual weeding days). Type 1, non-farm households; Type 2, part-time households; Type 3, livestock breed households; Type 4, subsidized households; Type 5, traditional planting households.

**Table 7:** Differences between household types regarding their cropland chemical disturbance behaviors (mean ± s.d.).

	Household Type				
	Type 1	Type 2	Type 3	Type 4	Type 5
Food crops (corn) planting					
NIF	665.13 ^ac^ ± 355.79	582.11 ^a^ ± 303.13	325.46 ^c^ ± 184.55	401.22 ^a^ ± 269.46	449.51 ^a^ ± 276.54
NIM	55.71 ^a^ ± 66.11	78.11 ^a^ ± 82.91	117.12 ^a^ ± 93.37	129.00 ^a^ ± 91.08	104.86 ^a^ ± 82.55
NI	720.85 ^a^ ± 382.21	660.22 ^a^ ± 286.55	442.58 ^a^ ± 202.88	530.22 ^a^ ± 290.86	554.37 ^a^ ± 246.99
PU	434.78 ^a^ ± 365.55	451.84 ^a^ ± 329.67	158.86 ^a^ ± 157.28	169.47 ^a^ ± 366.93	290.42 ^a^ ± 302.15
HU	350.01 ^a^ ± 226.95	464.63 ^b^ ± 258.84	248.96 ^a^ ± 154.75	160.46 ^bd^ ± 173.56	165.58 ^be^ ± 112.24
Cash crops (tobacco) planting					
NIF	167.83 ^a^ ± 24.06	128.97 ^a^ ± 34.17	100.55 ^ce^ ± 35.15	106.71 ^a^ ± 33.52	183.40 ^e^ ± 74.25
NIM	25.03 ^ac^ ± 25.03	30.01 ^a^ ± 15.12	38.46 ^a^ ± 14.13	43.22 ^a^ ± 20.24	48.12 ^c^ ± 37.03
NI	192.86 ^a^ ± 49.08	167.43 ^a^ ± 32.45	130.53 ^a^ ± 49.27	124.24 ^a^ ± 32.71	231.52 ^a^ ± 91.12
PU	550.30 ^a^ ± 50.30	769.23 ^a^ ± 223.72	766.60 ^a^ ± 44.35	539.1 ^a^ ± 37.4	768.14 ^a^ ± 298.99
HU	180.18 ^a^ ± 100.04	293.44 ^bd^ ± 70.56	221.42 ^a^ ± 120.27	151.82 ^d^ ± 66.30	271.32 ^a^ ± 276.43

Notes: The superscripts indicate significant between-group differences (Kruskal-Wallis test, *p* ≤ 0.05). Abbreviations are: NIF, total input of exogenous N from inorganic fertilizers; NIM, total input of exogenous N from farm manure; NI, total input of exogenous N from inorganic fertilizers and farm manure; PU, pesticides costs per hectare; HU, herbicides costs per hectare. Type 1, non-farm households; Type 2, part-time households; Type 3, livestock breed households; Type 4, subsidized households; Type 5, traditional planting households.

## Data Availability

The data presented in this study are available on request from the corresponding author.
